# Dimethyl fumarate abrogates striatal endoplasmic reticulum stress in experimentally induced late-stage Huntington’s disease: Focus on the IRE1α/JNK and PERK/CHOP trajectories

**DOI:** 10.3389/fphar.2023.1133863

**Published:** 2023-03-28

**Authors:** Lina Y. Hassab, Samah S. Abbas, Reham A. Mohammed, Dalaal M. Abdallah

**Affiliations:** ^1^ Department of Pharmacology and Toxicology, Faculty of Pharmacy, Misr International University, Cairo, Egypt; ^2^ Department of Pharmacology and Toxicology, Faculty of Pharmacy, Cairo University, Cairo, Egypt

**Keywords:** unfolded protein response, dopamine, AKT/mTOR and CREB/BDNF/TrkB, miR-634, ER stress/oxidative stress, apoptosis

## Abstract

**Introduction:** Dimethyl fumarate (DMF) is FDA-approved for use in patients with relapsing multiple sclerosis, and it processes neuroprotection in several experimental settings; however, its impact on combating Huntington’s disease (HD) remains elusive. This study aimed to explore the role of DMF post-treatment on HD mediated endoplasmic reticulum (ER) stress response in a selective striatal degeneration HD model.

**Methods:** Rats, exposed to 3-nitropropionic acid, were either left untreated or post-treated with DMF for 14 days.

**Results and Discussion:** DMF reduced locomotion deficits in both the open field and beam walk paradigms, boosted the striatal dopamine (DA) content, improved its architecture at the microscopic level, and hindered astrogliosis. Mechanistically, DMF limited the activation of two of the ER stress arms in the striatum by reducing p-IRE1α, p-JNK, and p-PERK protein expressions besides the CHOP/GADD153 content. Downstream from both ER stress arms’ suppression, DMF inhibited the intrinsic apoptotic pathway, as shown by the decrease in Bax and active caspase-3 while raising Bcl-2. DMF also decreased oxidative stress markers indicated by a decline in both reactive oxygen species and malondialdehyde while boosting glutathione. Meanwhile, it enhanced p-AKT to activate /phosphorylate mTOR and stimulate the CREB/BDNF/TrkB trajectory, which, in a positive feedforward loop, activates AKT again. DMF also downregulated the expression of miRNA-634, which negatively regulates AKT, to foster survival kinase activation.

**Conclusion:** This study features a focal novel point on the DMF therapeutic ability to reduce HD motor manifestations *via* its ability to enhance DA and suppress the IRE1α/JNK and PERK/CHOP/GADD153 hubs to inhibit the mitochondrial apoptotic pathway through activating the AKT/mTOR and BDNF/TrkB/AKT/CREB signaling pathways and abating miRNA-634 and oxidative stress.

## 1 Introduction

Huntington’s disease (HD) is a neurodegenerative autosomal dominant disease with the adult onset deteriorating over 10–20 years (Labbadia and Morimoto, 2013). It is characterized by a triad of manifestations that begins with chorea developing into dystonia besides psychiatric symptoms and a cognitive decline (André et al., 2010). With an ambiguous pathogenesis, HD is caused by mutant huntingtin (mHtt) protein that is prone to misfolding, leading to endoplasmic reticulum (ER) stress (Reijonen et al., 2008). HD is also associated with astroglial activation (Palpagama et al., 2019), neuro-inflammation (Rocha et al., 2016), mitochondrial dysfunction, destruction of dopaminergic neurons, and apoptotic events (McColgan and Tabrizi, 2018). However, a low level of brain-derived neurotrophic factor (BDNF) has also been linked to HD pathogenesis (Zuccato and Cattaneo, 2009; Mohamed et al., 2023).

In HD (Shacham et al., 2019) and other neurodegenerative diseases (Sprenkle et al., 2017), the accumulation of misfolded protein instigates the ER stress track that activates unfolded protein response (UPR) in an attempt to readjust the ER protein-folding capacity (Hetz et al., 2020). Among the UPR sensors responsible for activation of the elemental ER stress pathway are protein kinase RNA-like ER kinase (PERK; van Vliet et al., 2017) and inositol-requiring enzyme 1α (IRE1α; Adams et al., 2019). In tedious stressful conditions, the propitious consequences of IRE1α incitement can be hampered transiently, while PERK signaling inclines can become more prevalent (Romine and Wiseman, 2019). When ER stress is activated, protein translation constrains through phosphorylation of the α-subunit of eukaryotic translation initiation factor 2 (eIF2α; Baird and Wek, 2012), eventually arresting translation of mRNAs to decrease the protein misfolding burden. C/EBP homologous protein (CHOP/GADD153), an indirect downstream target of both PERK and IRE1α (Oyadomari and Mori, 2004), pushes the cell fate toward apoptosis (Hu et al., 2019). p-IRE1α is also involved in apoptotic cell death by prompting c-Jun N-terminal kinase (JNK) stimulation and spliced X-box binding protein 1 (XBP1) to produce the active transcription factor XBP1s (Li et al., 2018).

Dimethyl fumarate (DMF) is used to medicate relapsing-remitting multiple sclerosis (Saidu et al., 2019) upon hydrolysis into monomethyl fumarate (MMF). Fumarates are thiol-reactive electrophilic agents that directly activate the nuclear factor (erythroid-derived 2)-like 2 (Nrf2) pathway to mitigate cellular oxidative stress (Akino et al., 2019); in addition, the latter is indirectly activated by the survival kinase AKT, affording neuroprotection against tauopathy in a mouse model (Cuadrado et al., 2018). DMF also activates hydroxycarboxylic acid receptor 2 (HCAR2) to switch the pro-inflammatory phenotype to the neuroprotective microglia phenotype in a manner independent of Nrf2 (Parodi et al., 2021). Moreover, in hysterectomized rat models with Alzheimer’s disease (AD), this fumarate ester was able to inhibit astrogliosis (Abd El-Fatah et al., 2021). Indeed, DMF has a good therapeutic application in terms of antioxidant, anti-inflammatory, and anti-apoptotic effects (Ragab et al., 2020; Abd El-Fatah et al., 2021). However, to the best of our knowledge, the clear-cut process behind the DMF curative benefit against HD pathogenesis is not delineated yet. Accordingly, this study aimed to unveil the potential of DMF in ameliorating motor dysfunction associated with late-stage HD. The main focus is on PERK and IRE1α, two decisive arms of the ER stress response, besides the possible involvement of the BDNF/TrkB/AKT/CREB and AKT/mTOR signaling pathways as part of its therapeutic action.

## 2 Materials and methods

### 2.1 Chemicals

DMF was suspended in 0.08% carboxymethyl cellulose (CMC), and 3-nitropropionic acid (3-NP) was dissolved in saline; both were purchased from Sigma-Aldrich (MO, United States). All other chemicals were of analytical grade, and the used kits and antibodies sources are provided in the relevant methodology section.

### 2.2 Animals

Adult male Wistar rats (200–250 g) were obtained from the National Research Center (NRC, Giza, Egypt). The experimental conduct was held at the animal facility of the Faculty of Pharmacy, Misr International University (MIU, Cairo, Egypt). Rats were acclimatized for a week; they had access to food and water and were kept in standard housing conditions: humidity, 60% ± 10%; room temperature, 25 ± 2 ֯ C; and a light/dark cycle of 12/12 h before and during the experimental period.

### 2.3 Compliance with ethical standards

Attempts were made to limit the number of animals used and reduce their discomfort. The management of animals strictly adhered to the Guide for the Care and Use of Laboratory “ARRIVE guidelines” (Kilkenny et al., 2010), and the study was carried out in accordance and compliance with the National Institutes of Health Guide for the Care and Use of Laboratory Animals (NIH, revised 2011). The protocol was accepted by the Research Ethics Committee, Faculty of Pharmacy, Cairo University (Cairo, Egypt; PT2660).

### 2.4 Selective striatal degeneration HD model

Rats received a daily intraperitoneal injection of 3-NP (12 mg/kg/d) for 28 consecutive days to produce selective progressive striatal degeneration (late-stage HD model) (Brouillet et al., 1995).

### 2.5 Experimental design

**FIGURE 1:**
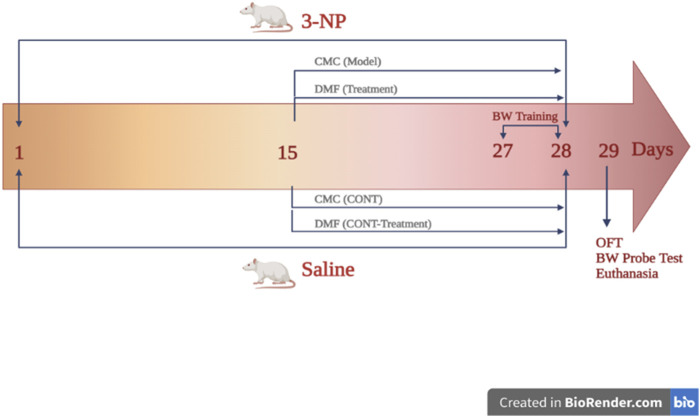
Outline of the experimental design showing the timeline for HD induction and DMF administration. On the first day, rats in groups (CONT) I and (DMF) II received saline until day 28; on the 15th day, group I rats additionally received CMC, whereas those in group II were administered DMF (25 mg/kg/d, p. o) for 2 weeks. Group (HD model) III and (HD + DMF) IV rats received 3-NP for 28 days with or without 2 weeks of DMF treatment that started on day 15. HD rats received oral CMC for 14 consecutive days initiated on day 15. On the 27th day, beam walk training was carried out for 2 days (3 trials/d/rat) and on day 29, OF and BW tests were performed; then, rats were sacrificed for sampling. BW, beam walk; CMC, carboxymethyl cellulose; CONT, control; DMF, dimethyl fumarate; HD, Huntington’s disease; 3-NP, 3-nitropropionic acid; OF, open field. The figure is created in BioRender.com.

Rats were randomly classified into four main groups (*n* = 9 rats each; Figure 1): group I and II animals received saline starting from day 1 to day 28. On day 15, rats in group I additionally received CMC to serve as the control (CONT) group, whereas those in group II were administered DMF (25 mg/kg/d, p. o; Ragab et al., 2020) for 2 weeks to be the DMF group. Rats in groups III (HD model) and IV (HD + DMF) received 3-NP. In the HD model group, rats were additionally administered oral CMC, the drug vehicle, from days 15 to 28, while in the HD + DMF group, animals received DMF for 2 weeks, starting on day 15 after development of HD symptoms.

### 2.6 Behavioral tests

Behavioral experiments were conducted 24 h after the last injection (Figure 1). For habituation, animals were conveyed to the testing facility, where they stayed at their cages for 1 h. Then, each rat was subjected to two behavioral experiments, beginning from the mild to the more stressful examination, i.e., open field examination, followed by the beam walk test. Both behavioral experiments were carried out in a dim light condition and videotaped for further analysis.

#### 2.6.1 Open field test

The open field test was used to examine the locomotor functions of animals. The open field arena has dimensions of 70 × 70 × 40 cm (length x width x height) with a gray polyvinyl chloride plastic board, and the floor was equally divided into squares with a center and peripheral regions. After habituation in their home cages, each animal was placed in the center area to explore the open field arena for 5 min; then, 70% ethanol was used to clean the arena between each animal. The latency time (the time taken to move from the center to the peripheral region) and ambulation frequency (the number of squares navigated by each animal) were recorded (Kraeuter et al., 2019).

#### 2.6.2 Beam walk test

Starting from day 27 of the experiment and following a 1 h habituation period, animals were individually brought to be trained on the beam apparatus three times for 2 days; then, the animals were tested on the third day (day 29) 2 h after performing the open field test. The apparatus consisted of a 1-m beam of 12 mm width and 50 cm height above the ground. The finish point consisted of a black box that had nesting material; the time was recorded from 0 cm of the beam and ended at 80 cm (Luong et al., 2011).

### 2.7 Sampling

After behavioral experiments, animals (*n* = 9 rats/group) were euthanized. Both striata of six rats/group were homogenized in PBS for colorimetric and ELISA assessments. Brains of the remaining three rats of the group were isolated, and one hemisphere was fixed in buffered formalin/saline (BFS) and embedded in paraffin wax blocks for screening of microscopic striatal alterations and immunochemical inspection. Notably, the striatum of the other hemisphere was isolated, split, and submerged in the corresponding suitable buffers for further Western blotting and qRT-PCR analyses. All samples were aliquoted and stored at −80°C until assessment, and their protein contents were assessed using the Bradford method (Bradford, 1976).

### 2.8 Western blot analysis of striatal p-AKT, p-CREB, p-TrkB, p-mTOR, p-PERK, p-IRE1α, and p-JNK

The protein abstraction kit (Millipore, MA, United States, Cat#: 2140) was used to quantify the amount of striatal protein in sample lysates; a similar load was detached by SDS-PAGE and electro-transferred to PVDF membranes that were blocked for 1 h in TBS-T20 buffer and 3% BSA at room temperature. The membranes were incubated overnight with phosphorylated antibodies (Thermo Fisher Scientific, MA, United States) of protein kinase B (*p*S473-AKT1; Cat#: PA5-85513), cAMP response element-binding protein (*p*S133-CREB; Cat#: PA5-85645), tropomyosin receptor kinase B (*p*Y515-TrkB; Cat#: PA5-105013), mammalian target of rapamycin (*p*S2448-mTOR; Cat#: 44-1125G), PERK (*p*T982-PERK; Cat# PA5–40294), IRE1α (*p*S724-IRE1α; Cat#: PA1–16927), and JNK (*p*T183/T185-JNK1/JNK2; Cat#: 44-682G) besides the antibody for the housekeeping gene β-actin purchased from Invitrogen Life Technologies (Scotland, United Kingdom). This was succeeded by overnight incubation with the horseradish peroxidase-conjugated rabbit anti-mouse secondary polyclonal antibody (Thermo Fisher Scientific; Cat#: 61-6520) at 4°C, followed by chemiluminescence detection (WesternBreeze Chemiluminescent Kit; Thermo Fisher Scientific; Cat#: WB7106). The estimated parameters were subjected to densitometric analysis using Bio-Rad software (CA, United States) for quantification relevant to the housekeeping gene.

### 2.9 qRT-PCR determination of striatal miRNA-634 expression

The striatum underwent total RNA extraction (TRIzol, Invitrogen, CA, United States) according to the provided instructions. Spectrophotometer (A260/A280 nm) was used to confirm the purity of the acquired RNA. The Path-ID™ Multiplex One-Step RT-PCR Kit (Thermo Fisher Scientific, Cat#: 4442136) was used to reversibly transcribe equivalent quantities of the isolated intact RNA to cDNA, according to the provided instructions. MicroRNA-634 (miRNA-634) was quantified by applying SYBR Green JumpStart Taq ReadyMix (Sigma-Aldrich) according to the constructor’s protocol. A measure of 5 µL of cDNA was combined with 12.5 uL of SYBR Green mixture, 5.5 uL of RNase-free water, and 2 uL of primers. The forward (5′-CAG​TCT​CAA​ACC​AGC​ACC-3′) and reverse (5′-TAT​GGT​TGT​TCA​CGA​CTC​CTT​CAC-3′; U6) primer sequence sets were used. Amplification of PCR was accomplished by 15 s of denaturation at 95°C for 40 cycles, 60 s of annealing at 60°C, and 60 s of extension at 72°C. The expression of the relative miRNA-634 levels against RNU6B was achieved using the 2^−ΔΔCT^ method (Livak and Schmittgen, 2001).

### 2.10 Quantification of striatal contents of CHOP/GADD153, active caspase-3, ROS, MDA, GSH, DA, BDNF, Bcl-2, and Bax by ELISA

The striatal contents of CHOP/GADD153 (Cat#: MBS3808179), active caspase-3 (Cat#: MBS7244630), reactive oxygen species (ROS; Cat#: MBS039665), malondialdehyde (MDA; Cat#: MBS8807536), glutathione (GSH; Cat#: MBS8807501), and B-cell lymphoma-2 (Bcl-2; Cat# MBS2881713) were analyzed using the Rat ELISA kit purchased from MyBioSource (CA, United States), while those of dopamine (DA; Cat#: ab285238) and BDNF (Cat#: ab213899) were assessed using Rat ELISA kits purchased from Abcam (Cambridge, United Kingdom). The striatal content of B-cell lymphoma-2-associated X (Bax; Cat# LS-F21494) was assessed by the Rat ELISA kit purchased from LifeSpan BioSciences (WA, United States). All assessments were performed according to the manufacturers’ instructions.

### 2.11 Hematoxylin and eosin (H&E) and Nissl staining

Sagittal 4-μm-thick sections were cut by a rotatory microtome for demonstration of striatal regions in different samples and analyzed in a blinded fashion. For generic morphological diagnosis, tissue sections were stained by H&E using a standard method that was followed by ordinal scoring for assessment of tissue damage in six non-overlapping random areas, where 0, 1, 2, and 3 are numbers assigned to normal, mild, moderate, and severe injuries (Stevens, 1946). For the quantitative inspection of the intact cell count, the toluidine blue staining method was used and the six non-overlapping random areas were analyzed and inspected for the intact neuronal mean count in the striatum. The micrographs and data were attained using the Leica application module for histological examination (Leica Microsystems GmbH, Wetzlar, Germany).

### 2.12 Striatal GFAP immunoreactivity

Immunohistochemical detection of glial fibrillary acidic protein (GFAP) within the striatum was carried out using the avidin–biotin complex (ABC) method. Sections were dewaxed, dehydrated, and incubated with the rat anti-GFAP antibody purchased from Thermo Fischer Scientific (Cat#13–0300; 1:200). Afterward, PBS-washed sections were incubated with the secondary antibody HRP EnVision kit (Dako, CA, United States). The bound antibody was visualized using the commercial ABC (Santa Cruz Biotech, CA, United States) system with chromogen 3,3′-diaminobenzidine (DAB) tetrahydrochloride, followed by hematoxylin as counter stain. The GFAP-positive reaction was observed by brown coloration of astrocytes, including their bodies, and processes and the GFAP percentage of immunoreactivity were calculated from the average of six non-overlapping fields.

### 2.13 Statistical analysis

The data were first analyzed for normality and homogeneity by Shapiro–Wilk and Bartlett’s tests, whenever applicable, and then conveyed as the mean ± SD or median and the range with first and third quartiles using GraphPad Prism v.9.0 (CA, United States). The data were analyzed by multiple comparisons by one-way analysis of variance (ANOVA), followed by Tukey’s *post hoc* test for parametric measurements, whereas the Kruskal–Wallis test followed by Dunn’s test was applied to the non-parametric data; both were applied with a fixed level of significance at *p* < 0.05.

## 3 Results

As DMF treatment in normal rats did not show any significant difference compared to that in CONT animals receiving the vehicles, therefore all comparisons were only conducted against the CONT group.

### 3.1 DMF improves locomotion and enhances DA in HD rats

**FIGURE 2:**
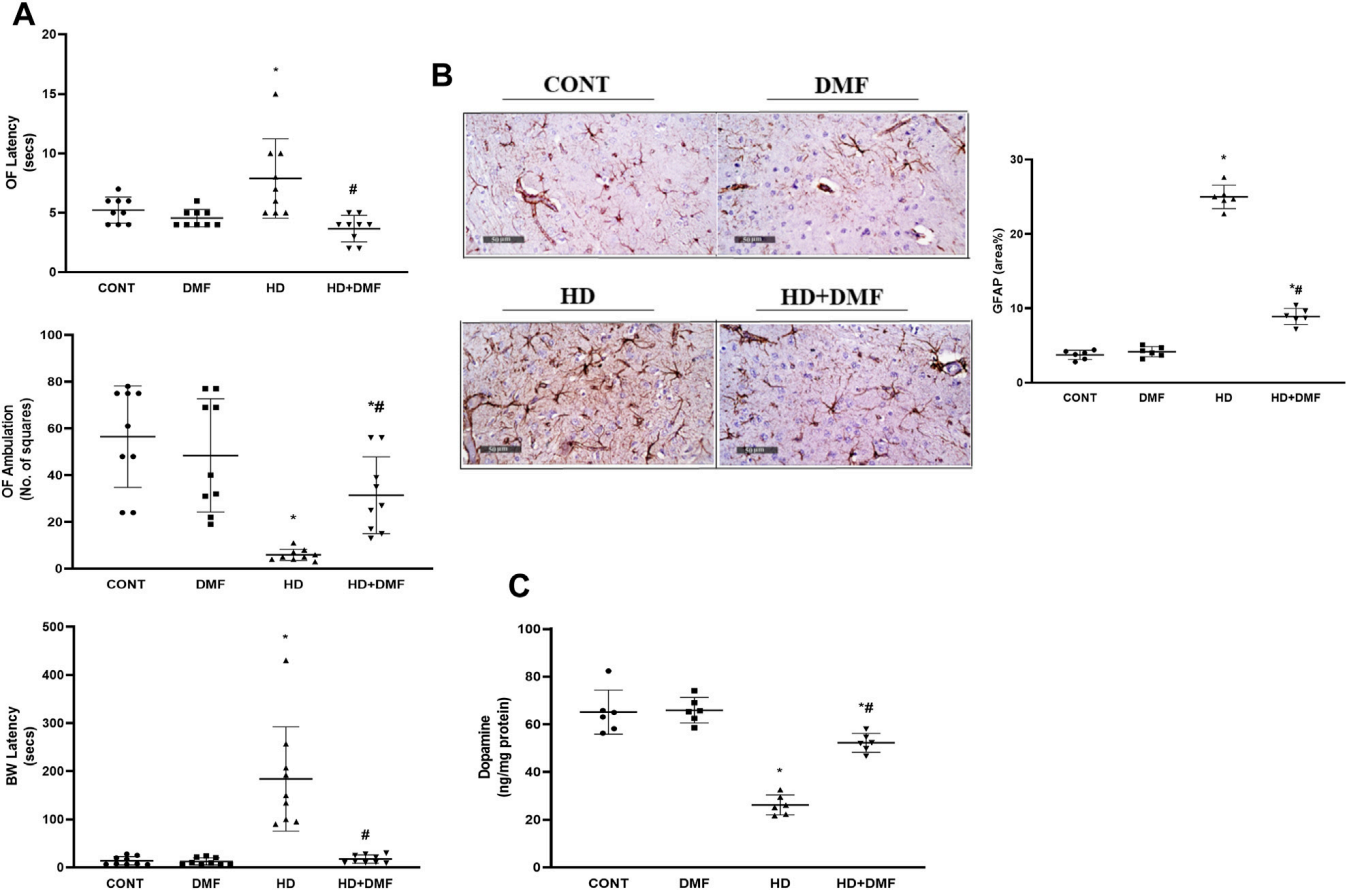
Effect of DMF on **(A)** latency time/ambulation frequency in the OF and BW latency to platform; **(B)** GFAP immunoreactivity in striatum and its quantitative analysis; **(C)** DA content in the striatum of HD rats. Data (six non-overlapping fields; *n* = 3 rats/group for GFAP; *n* = 6 rats/group for ELISA; n = 9 rats/group for OF, BW) are represented as mean ± SD and analyzed by one-way ANOVA, followed by Tukey’s *post hoc* test; * vs. CONT and # vs. HD using at *p* < 0.05. BW, beam walk; CONT, control; DA, dopamine; DMF, dimethyl fumarate; GFAP, glial fibrillary acidic protein; HD, Huntington’s disease; OF, open field.

As represented in Figure 2A, HD rats showed impaired locomotion in the open-field test evidenced by the 1.5-fold increase in the latency with 90% decrease in the ambulation frequency *versus* CONT. In association, dysfunctional motor coordination was noted as 13.3-fold higher latency to cross the beam in HD animals. However, DMF succeeded to restore the motor function by evading muscle rigidity/coordination to improve locomotion. This could be linked directly to the drug’s ability to (B) reduce striatal GFAP immunoreactivity to 36% and (C) enhance the striatal content of DA (2 folds), the main predictor for adequate motor performance, which was deterred upon 28 days of prolonged 3-NP exposure (40%).

### 3.2 DMF ameliorates 3-NP-induced neurodegeneration in the striatum

**FIGURE 3:**
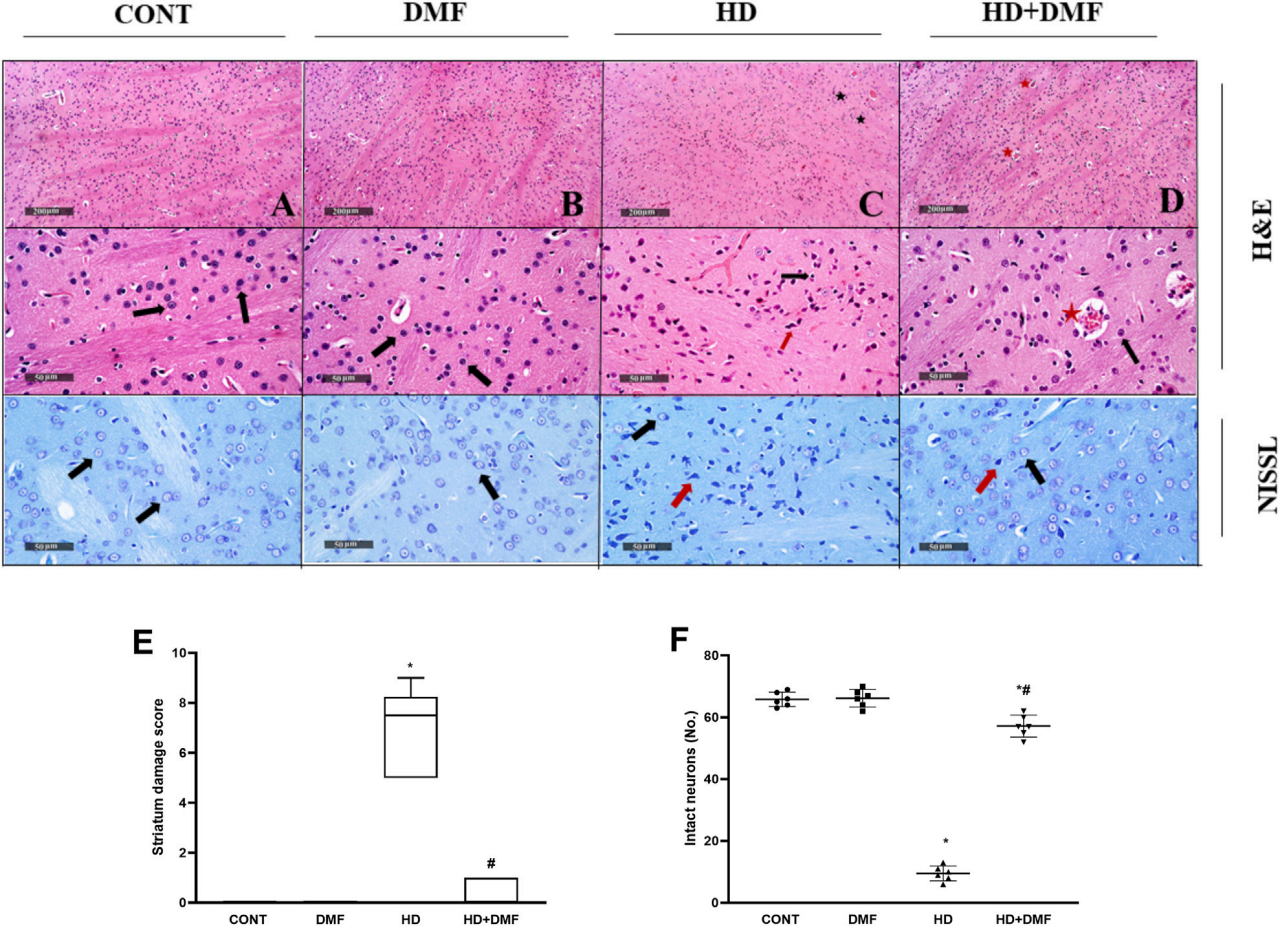
Effect of DMF on striatal histological changes induced by HD. **(A)** CONT micrograph showing ordinary histological architecture *(black arrow*s); **(B)** DMF showing no abnormal findings; **(C)** HD showing bountiful degeneration of neurons with contracted pyknotic neuronal cell bodies *(red arrow),* moderate blood vessel congestion *(star)*, and slight infiltration of focal glial cells *(black arrow* ***)***; **(D)** HD + DMF showing marked improvement of morphological features with intact neurons and subcellular components *(black arrow)* with moderately congested blood vessels *(star)*. The data (six non-overlapping fields; *n* = 3 rats/group) in panel **(E)** represent the striatum damage score that is represented as dot boxplots displaying minimum, maximum, median, and the first and third quartiles, and statistically analyzed by non-parametric Kruskal–Wallis and Dunn’s *post hoc* tests, whereas the panel **(F)** presents a mean number of Nissl-stained cells (intact neurons) ± SD tested for significance using one-way ANOVA, followed by Tukey’s *post hoc* test; * vs. CONT and # vs. HD at *p* < 0.05. CONT, control; DMF, dimethyl fumarate; HD, Huntington’s disease.

As shown in Figure 3, both (A,E) CONT and (B,E) DMF rats showed normal striatal architecture and (F) intact neurons without any injury. A significant number of (C,E) shrunken pyknotic neuronal cell bodies, blood vessel congestion, and infiltration of focal glial cells, with the increased striatal median damage injury score to reach 7.5, were noted in HD rats. This was associated with (F) reduced Nissl-stained intact neurons signifying marked neuronal cell death in the 3-NP group. On the other hand, rats post-treated with DMF showed (D,E) mildly congested blood vessels, significantly reduced neuronal loss with a reduced injury score almost comparable to that of the CONT animals, and (F) an increased number of intact neurons indicative of survival.

### 3.3 DMF activates mTOR and shuts off striatal PERK and IRE1α ER stress arms to halt intrinsic apoptosis in HD rats

**FIGURE 4:**
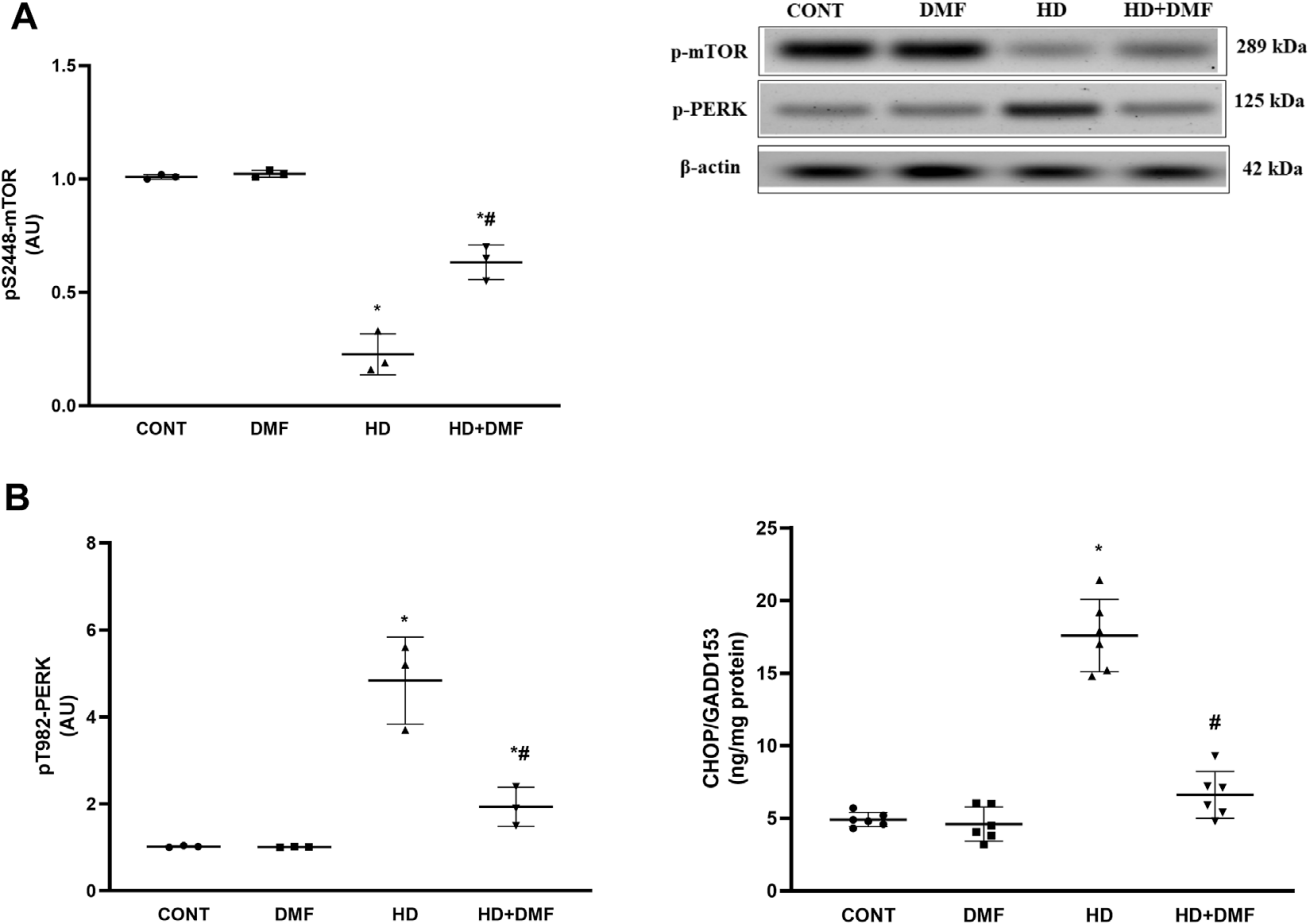
Effect of DMF on the striatal protein expression/content of **(A)** p-mTOR, **(B)** p-PERK, and CHOP/GADD153 in HD rats. Data are represented as mean ± SD (*n* = 3 rats/group for WB; *n* = 6 rats/group for ELISA) using one-way ANOVA, followed by Tukey’s *post hoc* test; * vs. CONT and # vs. HD at *p* < 0.05. CHOP/GADD153, C/EBP homologous protein; CONT, control; DMF, dimethyl fumarate; HD, Huntington’s disease; mTOR, mammalian target of rapamycin; PERK, protein kinase R-like endoplasmic reticulum kinase.

**FIGURE 5:**
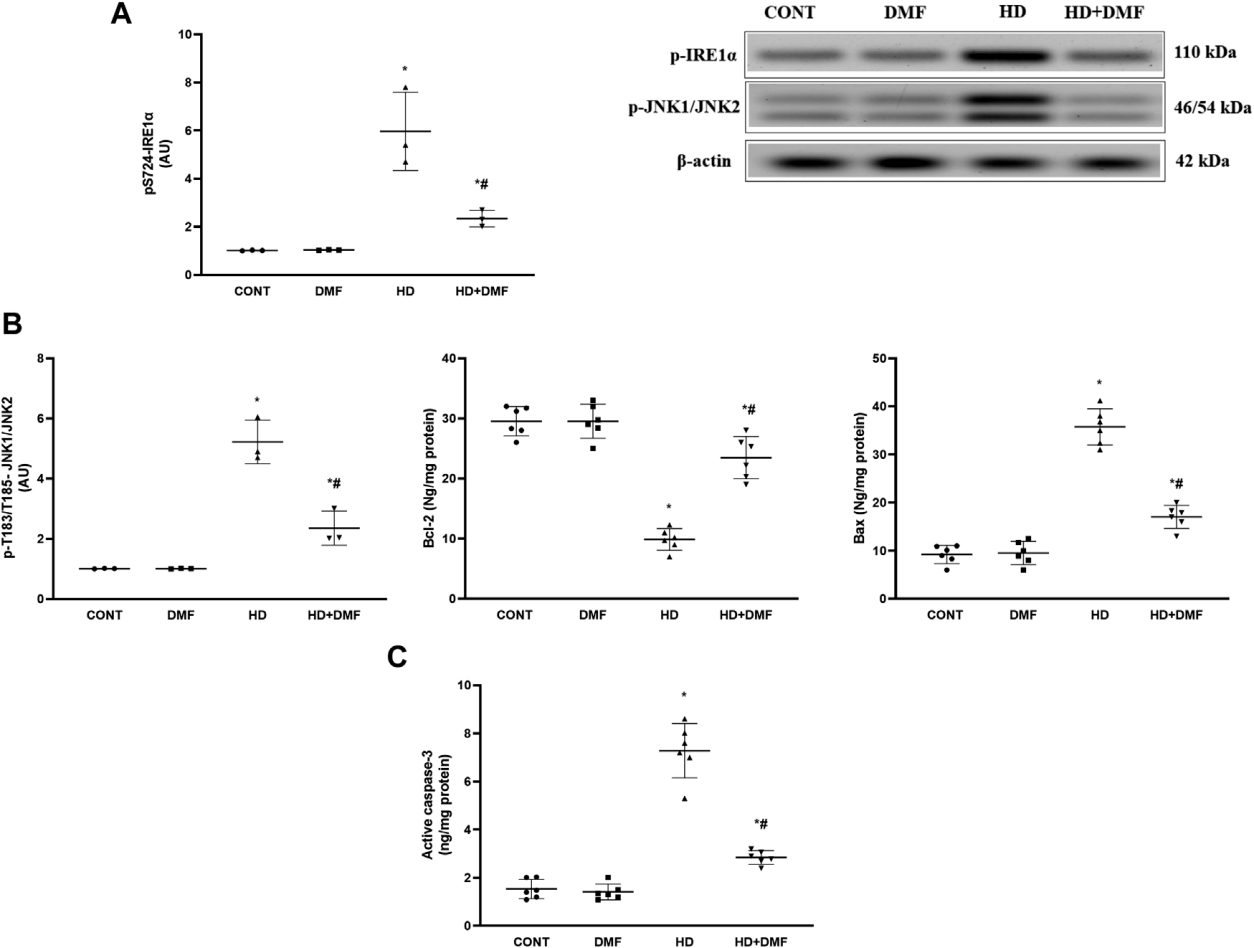
Effect of DMF on the striatal protein expression/content of **(A)** p-IRE1α; **(B)** p-JNK1/JNK2, Bcl-2, and Bax; and **(C)** active caspase-3 in HD rats. Data are represented as mean ± SD (*n* = 3 rats/group for WB; n = 6 rats/group for ELISA) using one-way ANOVA, followed by Tukey’s *post hoc* test * vs. CONT and # vs. HD at *p* < 0.05. Bax, B-cell lymphoma-2-associated X; Bcl-2, B-cell lymphoma-2; CONT, control; DMF, dimethyl fumarate; HD, Huntington’s disease; IRE1α, inositol-requiring enzyme 1 alpha; JNK, c-Jun NH2-terminal kinase.

During conditions where ER stress is prolonged, mTORC1 is inactivated or dephosphorylated to blunt its survival signal and the UPR is switched to a pro-apoptotic rather than a pro-survival module. As shown in Figure 4, 3-NP receiving rats showed (A) 78% decline in the striatal active *p*S2448-mTOR protein expression paralleled with (B) 4.7-fold increase in *p*T982-PERK protein expression and 3.6-fold elevation in the CHOP/GADD153 content in comparison with the CONT group. In Figure 5, HD induction (A) activated the most conservative UPR sensor, IRE1α, evidenced by 5.9-fold protein expression of its phospho-Ser724 isoform to boost (B) *p*T183/T185-JNK1/JNK2 (5.1-fold) and reduce Bcl-2 by 67%. This was accompanied by 3.8-fold and 4.7-fold respective increases in Bax and (C) active caspase-3 in comparison with the CONT group. Notably, DMF potently abrogated apoptosis *via* inhibiting the PERK/CHOP/GADD153 (Figure 4) and IRE1α/JNK tracks to reduce apoptosis (Figure 5) and microscopic alterations (Figure 3), thus improving motor performance of HD (Figure 2).

### 3.4 DMF activates the AKT trajectory *via* modulating miRNA-634, oxidative stress, and the BDNF hub in the striatum of HD rats

**FIGURE 6:**
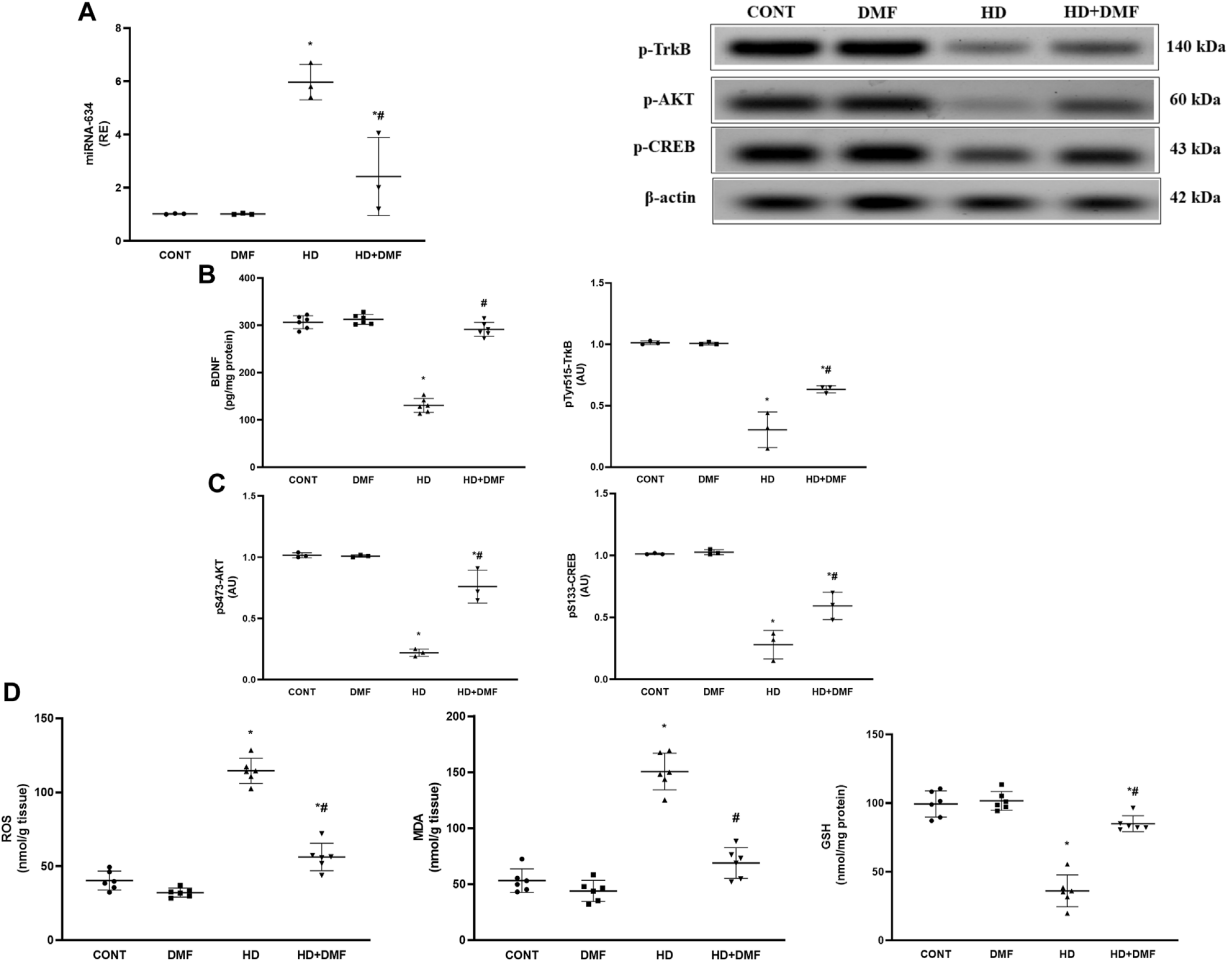
Effect of DMF on striatal **(A)** miRNA-634 expression; content/protein expression of **(B)** BDNF and p-TrkB; protein expression of **(C)** p-AKT and p-CREB; and content of **(D)** MDA, ROS, and GSH in the striatum of HD rats. Data are represented as mean ± SD (*n* = 3 rats/group for PCR,WB; *n* = 6 rats/group for ELISA) using one-way ANOVA, followed by Tukey’s *post hoc* test * vs. CONT and # vs. HD at *p* < 0.05. AKT, protein kinase B; BDNF, brain-derived neurotrophic factor; CONT, control; CREB, cAMP response element-binding protein; DMF, dimethyl fumarate; GSH, glutathione; HD, Huntington’s disease; MDA, malondialdehyde, miRNA-634, microRNA-634; ROS, reactive oxygen species; TrkB, tropomyosin receptor kinase B.

BDNF stimulates AKT to reduce oxidative stress reactions that mediate programmed cell death directly and indirectly *via* ER stress, whereas miRNA-634 acts as a negative regulator for AKT. As shown in Figure 6, 3-NP produced (A) 5.9-fold-upregulated striatal expression in miRNA-634 concurrent to the decreased contents/protein expressions of (B) the neurotrophic factor BDNF (42%), *p*Y515-TrkB (30%), (C) *p*S473-AKT (21%), and *p*S133-CREB (27%) compared to their CONT counterparts. In contrast to the diseased rats, DMF post-treatment activated the AKT/CREB trajectory by augmenting BDNF to activate/phosphorylate TrkB and *via* downregulating miRNA-634. Furthermore, (D) 2.8-fold increases of both striatal ROS and MDA contents with declined GSH to reach about the third CONT value were noticed, following 3-NP exposure, effects that were hampered *via* DMF post-administration.

## 4 Discussion

**FIGURE 7:**
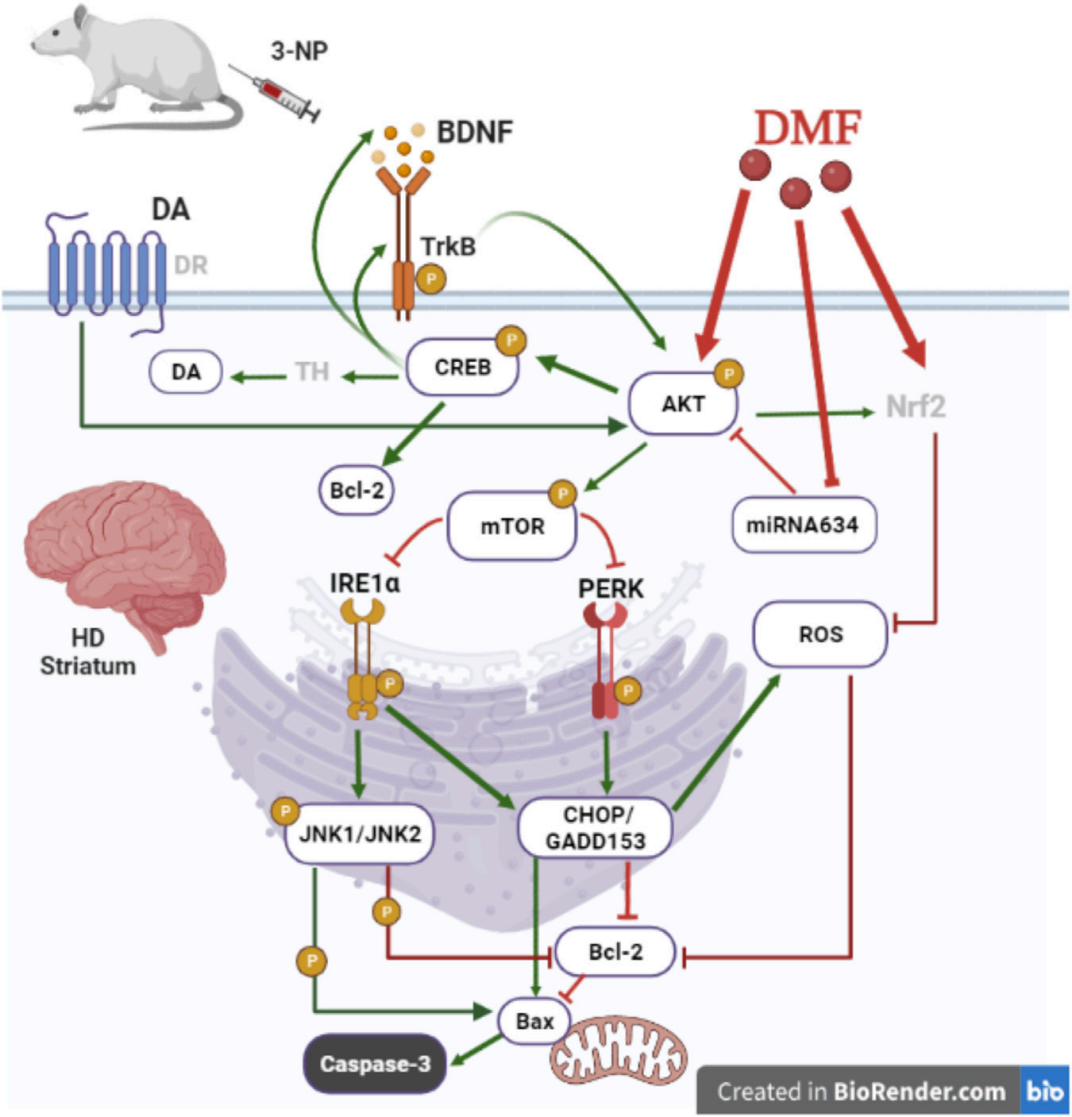
Proposed mechanism for DMF therapeutic efficacy in managing HD-induced motor dysfunction. DMF ameliorates striatal loss of neurons in a 3-NP induced HD model by 1) inducing AKT/mTOR activation to inhibit intrinsic apoptosis caused by both ER stress arms IRE1α/JNK and PERK/CHOP/GADD153 activation; 2) activating the BDNF/TrkB/AKT/CREB axis to overweigh the pro-survival Bcl-2 and enhance DA formation; 3) downregulating miRNA-634 to initiate AKT activation; and 4) suppressing the oxidative stress response consequent to CHOP/GADD153 inhibition to additionally suppress intrinsic apoptosis. AKT, protein kinase B, Bax, B-cell lymphoma-2-associated X; Bcl-2, B-cell lymphoma-2; BDNF, brain-derived neurotrophic factor; CHOP/GADD153, C/EBP homologous protein; CREB, cAMP response element-binding protein; DA, dopamine; DMF, dimethyl fumarate; DR, dopamine receptor; HD, Huntington’s disease; IRE1α, inositol-requiring enzyme 1 alpha; JNK, c-Jun NH2-terminal kinase; miRNA-634, microRNA-634; mTOR, mammalian target of rapamycin; 3-NP, 3-nitropropionic acid; Nrf2, nuclear factor (erythroid-derived 2)-like 2; PERK, protein kinase R-like endoplasmic reticulum kinase; ROS, reactive oxygen species; TH, tyrosine hydroxylase; TrkB, tropomyosin receptor kinase B. This figure is created in BioRender.com.

The present study sheds light on the novel therapeutic efficacy of DMF on managing motor hypofunction induced by degenerative striatal damage associated with late-stage HD. DMF hindered the loss of DA in the striatum, the main affected CNS region, which revealed more intact neurons accompanied with less microscopic alterations and reduced astrogliosis, signifying medium-sized spiny neuron preservation (Ciesielska et al., 2009; Hirsch et al., 2012). The drug primarily acted through activating/phosphorylating AKT/mTOR to deactivate both UPR response IRE1α and PERK arms, repressing their downstream targets, JNK and CHOP/GADD153, which drive caspase-3 activation *via* tipping the Bcl-2/Bax balance away from apoptosis. AKT/mTOR survival machinery was stimulated by several upstream signals, including DA and the CREB/BDNF/TrkB trajectory, in addition to miRNA-634 inhibition. DMF-mediated AKT/CREB active phosphorylation with CHOP/GADD153 and JNK inhibition reduced oxidative stress that also triggers intrinsic apoptosis (Figure 7).

Our study highlighted the role of DMF in attenuation of p-IRE1α, first reported in this study, considering it as a novel target for managing HD-mediated motor dysfunction. Previously, DMF blunted the formation of chaperone-binding immunoglobulin protein (BiP/GRP78), an ER stress marker, in an intervertebral disc degeneration *in vivo* model and *in vitro* using human nucleus pulposus cells (Zhu et al., 2020; Wang et al., 2021) to support its ability to alleviate the ER stress response. Notably, AKT activation/phosphorylation causes mTORC1 activation *via* phosphorylation of mTOR at Ser2448 that sustains a positive feedback loop for AKT activation (Navé et al., 1999; Cheng et al., 2019). Indeed, both verities were proven with the current DMF treatment in the striatum of HD rats to align with the activation/phosphorylation of AKT in neurodegenerative models (Cuadrado et al., 2018; Abd El-Fatah et al., 2021); however, this is the first report to verify the stimulatory effect of the drug on mTOR. AKT/mTOR is one axis that coordinates cell survival through cessation of ER stress by deactivating the IRE1α trajectory (Sanchez-Alvarez et al., 2017; Zaky et al., 2021). The current inactivation of IRE1α by DMF was associated with the suppression of p-JNK, CHOP/GADD153, pro-apoptotic Bax, and active caspase-3, as well as the enhancement of anti-apoptotic Bcl-2. Of note, CHOP/GADD153 is a transcription factor that directly controls apoptosis *via* upregulating Bax and downregulating Bcl-2 gene expression besides an indirect AKT-dependent mechanism (Hu et al., 2019). The coordinated phosphorylation of Bcl-2 and Bax by p-JNK, initiated by the activation of the ER stress IRE1α arm, mediates the intrinsic apoptotic response by allowing mitochondrial outer membrane permeabilization to activate caspase-3 (Wei et al., 2008; Verfaillie et al., 2013). Meanwhile, under the persistent activation of IRE1α, CHOP/GADD153 indirectly, by preventing AKT, eventually assists caspase-3 activation (Li et al., 2018; Hu et al., 2019) and Bax conformational changes, allowing its redistribution to the mitochondrial membrane to signal for apoptosis execution (Yamaguchi and Wang, 2001). On the other hand, AKT is also responsible for the transcription factor CREB active Ser-133 phosphorylation, which increases Bcl-2 gene expression (Tao et al., 1998; Finkbeiner, 2000), as seen with the current DMF treatment to shift the cell fate away from apoptosis. Additionally, BDNF, another downstream target of CREB, activates mTOR to enhance neuronal survival (Mamounas et al., 1995) *via* its interaction with the TrkB receptor that is also transcribed by CREB (Takei et al., 2004; Esvald et al., 2020). Notably in HD, BDNF (Wu et al., 2010), the utmost premeditated brain neurotrophin, and its receptor TrkB play prominent roles in neuronal survival (Ahmed et al., 2021), whereas BDNF deficiency is linked to HD progression (Ou et al., 2021). Although we have proven an anti-apoptotic role for DMF associated with BDNF striatal elevation, Smith et al. (2014) concluded that the neurotropic factor mediated neuronal survival through activating mTOR-dependent autophagy rather than caspase-3 inhibition or the upregulation of protein synthesis in primary rat hippocampal neurons. In convenience with our data, the anti-apoptotic role of DMF has been previously reported in a rat pentylenetetrazole kindling model (Singh et al., 2019). Other studies demonstrated the DMF capacity to suppress CHOP/GADD153 and Bax/caspase-3 with mutual Bcl-2 promotion in nucleus pulposus cells (Zhu et al., 2020; Wang et al., 2021). It also inhibited p-JNK and caspase-3 activity in the liver of depressed rats (Kortam et al., 2021) and activated the BDNT/TrkB/AKT/CREB hub in an AD model (Abd El-Fatah et al., 2021). The present findings may thus highlight the importance of DMF-induced AKT/mTOR and AKT/CREB activation in deactivating the IRE1α trajectory and accordingly the mitochondrial apoptotic cascade, thereby directing the UPR to the survival mode to prevent striatal neuronal loss and improve locomotion.

The second UPR arm PERK shut off by DMF can also justify the present decrease in CHOP/GADD153 to additionally repress the intrinsic apoptotic cascade since PERK enhances both the transcription and translation of CHOP/GADD153 (Rozpedek et al., 2016) to augment the IRE1α-death signal. In an *in vivo* cecal ligation/puncture model (Zaky et al., 2020), a shift of ER response toward apoptosis, following mTORC1 downregulation/PERK phosphorylation, has been reported, which is in a similar context to the currently used HD model. Therefore, the upregulation/phosphorylation of mTOR that also exerts inhibitory effects on the PERK axis, as previously documented in an *in vitro* study on gastrointestinal neuroendocrine cell lines (Freis et al., 2017), could afford a clarification for DMF anti-apoptotic potential through PERK-mediated CHOP/GADD153 suppression. By an additional survival mechanism, the deactivation of PERK, by DMF, can eventually restore global protein translation to annul its cutback in tedious conditions (Kaur et al., 2008).

ROS overproduction is considered a key element in the pathogenesis of HD being an additional reason for activating the intrinsic apoptotic pathway (Lu et al., 2019). These extremely reactive moieties also aggravate protein misfolding to detach the ER stress sensors from the ER chaperones and activate JNK to augment caspase-3 activation through the mitochondrial apoptotic pathway (Li et al., 2004; Stack et al., 2008; Shin et al., 2009; Liu et al., 2013; Chong et al., 2017; Chen et al., 2019). CHOP/GADD153 also spikes cellular oxidative stress by increasing NADPH oxidase (Chou et al., 2013). Indeed, a pro-oxidant status of the striatum in HD rats *versus* an antioxidant status after DMF treatment was demonstrated in our study. In the latter context, the drug suppressed astrogliosis, ROS, and MDA and replenished GSH. DMF, by acting directly as an Nrf2 activator, boosts the formation of antioxidant elements to reduce ROS formation as its basic mechanism (Wang et al., 2021). Once again, the activation of AKT by DMF could lend further credit to its antioxidant effect since Nrf2 is an additional transcriptional target of CREB (Katoh et al., 2001). BDNF was shown to attenuate the spike in ROS to enhance the survival of adult spinal cord-derived neural stem/progenitor cells (Hachem et al., 2015) and foster antioxidant responses in primary hippocampal cultures through Nrf2 transcriptional activity (Bruna et al., 2018), suggesting that the DMF antioxidant activity can also stem from BDNF/TrkB/AKT/CREB axis activation. In support to our findings, DMF boosted the level of BDNF in a spinal cord injury model (Cordaro et al., 2017) and hypothyroid rat brain (Pan et al., 2022), alleviated depression-like symptoms through BDNF upregulation (Abd El-Fattah et al., 2018), and activated the TrkB/AKT/CREB trajectory in association with reduced ROS production in an AD model (Abd El-Fatah et al., 2021). Therefore, BDNF, through fortifying antioxidant enzymes, could potentiate DMF antioxidant armor by counteracting ROS activation of the mitochondrial apoptotic pathway besides the inactivation of the PERK and IRE1α hubs.

Notably, other routes of the pro-survival kinase AKT activation include the action of DA that was enhanced, following DMF administration. In this regard, Swift et al. (2011) reported that both D1R activation and D2R activation can trans-activate the BDNF receptor in neurons and the treatment with the D2R agonist led to marked elevation in AKT phosphorylation, as previously depicted in an *in vitro* study of neuronal cell culture model system (Brami-Cherrier et al., 2002). The present enhancement of DA could be related to the increase in BDNF since it dynamizes endurance and continuity of DA neurons (Razgado-Hernandez et al., 2015), increases the number of existing DA receptors and tyrosine hydroxylase responsible for DA production through activating CREB (Baquet et al., 2005; Chen et al., 2021), and expands the DA transporter uptake potential (Ziebell et al., 2012), all which leads to reinforcing the DA action to hence highlight the pivotal role played by DMF in orchestrating neuronal survival that facilitates locomotion.

miRNAs are considered non-coding small molecules of RNA that are heavily located in the CNS (Wang et al., 2014). They have received widespread interest for their pivotal roles in different cell survival trajectories besides cell apoptosis (Berezikov and Plasterk, 2005). It is important to note that miRNA-634 has a sequence like human PI3KR1, which synchronizes the PI3K regulatory subunit (Cui et al., 2016), where its downregulation plays a crucial role in hindering neuronal apoptosis by directly impacting PI3K/AKT phosphorylation (Chang et al., 2018). This is in accordance with our findings, where the DMF-treated group experienced significant reduction in miRNA-634 expression in comparison with the insult to give a further justification for DMF anti-apoptotic potential and further credit to the drug survival machinery.

To this end, DMF, by suppressing pro-apoptotic action induced by UPR, has led to preservation of the DA function to improve locomotor behavior and deter microscopic striatal alterations. This is mediated by inhibition of the two principal UPR receptors, PERK and IRE1α, followed by blunting CHOP/GADD153 and JNK apoptotic upstream. Both the ER stress arms are downregulated as a direct result of AKT/mTOR trajectory activation and indirectly by inhibition of oxidative stress and miRNA-634 in credit to AKT/CREB/BDNF/TrkB activation besides the direct stimulatory effect of the drug to preserve DA function. Based on the reported data, DMF could be endorsed as a future therapeutic mark for attenuating HD complications through amending the UPR/ER stress response equilibrium.

## Data Availability

The original contributions presented in the study are included in the article/Supplementary Material; further inquiries can be directed to the corresponding author.
